# Sympathetic components in left and right human cervical vagus nerve: implications for vagus nerve stimulation

**DOI:** 10.3389/fnana.2023.1205660

**Published:** 2023-07-10

**Authors:** Tom J. H. Ruigrok, Sophia A. Mantel, Lara Orlandini, Corné de Knegt, Arnaud J. P. E. Vincent, Jochem K. H. Spoor

**Affiliations:** ^1^Department of Neuroscience, Erasmus MC, University Medical Center, Rotterdam, Netherlands; ^2^Department of Neurosurgery, Erasmus MC, University Medical Center, Rotterdam, Netherlands

**Keywords:** vagus nerve, tyrosine hydroxylase, autonomous nervous system, superior cervical ganglion, sympathetic trunk

## Abstract

Cervical vagus nerve stimulation is in a great variety of clinical situations indicated as a form of treatment. It is textbook knowledge that at the cervical level the vagus nerve contains many different fiber classes. Yet, recently, several reports have shown that this nerve also may contain an additional class of potentially noradrenergic fibers, suggested to denote efferent sympathetic fibers. As such, the nature and presence of these fibers should be considered when choosing a stimulation protocol. We have studied human vagus material extracted from dissection room cadavers in order to further confirm the presence of this class of fibers, to study their origin and direction within the nerve and to determine their distribution and variability between subjects and pairs of left and right nerves of the same individual. Sections were studied with immunohistochemical techniques using antibodies against tyrosine hydroxylase (TH: presumed to indicate noradrenergic fibers), myelin basic protein and neurofilament. Our results show that at least part of the TH-positive fibers derive from the superior cervical ganglion or sympathetic trunk, do not follow a cranial but take a peripheral course through the nerve. The portion of TH-positive fibers is highly variable between individuals but also between the left and right pairs of the same individual. TH-positive fibers can distribute and wander throughout the fascicles but maintain a generally clustered appearance. The fraction of TH-positive fibers generally diminishes in the left cervical vagus nerve when moving in a caudal direction but remains more constant in the right nerve. These results may help to determine optimal stimulation parameters for cervical vagus stimulation in clinical settings.

## Introduction

Invasive and non-invasive stimulation of the cervical vagus nerve is used to modulate a great variety of conditions such as epileptic seizures (Muthiah et al., 2023), depression (Theiss and Slavin, 2022), gastrointestinal disorders (Payne et al., 2019), inflammation (Alvarez et al., 2022), heart failure (Konstam et al., 2022), migraine and other headaches (Straube and Eren, 2021). Hence, as the vagus nerve, especially in its cervical course, contains several groups of fibers, it is vital to understand what actually is stimulated by cervical vagus stimulation and how its effect is induced. Classically, the cervical vagus nerve is reported as containing (1) efferent fibers for parasympathetic innervation of organs, originating from the dorsal vagal motor nucleus and ambiguus nucleus (general visceromotor fibers), (2) branchiomotor fibers innervating pharynx and larynx muscles, originating from the ambiguus nucleus (special visceromotor fibers), (3) general somatosensory afferent fibers that terminate in the spinal trigeminal nucleus, (4) general viscerosensory afferent fibers from the organs that terminate in the caudal part of the nucleus of the solitary tract and (5) special viscerosensory afferents that represent taste fibers from the back of the tongue and lower pharynx that terminate in the rostral part of the nucleus of the solitary tract (Saper et al., 2013).

However, several reports have indicated that the human vagus nerve also contains catecholaminergic fibers. Catecholaminergic fibers can be recognized by their positive staining for tyrosine hydroxylase, which catalyses the conversion of L-tyrosine to L-3,4-dihydroxyphenylalanine (L-DOPA), which is a precursor for the catecholaminergic transmitters dopamine and noradrenaline (norepinephrine). None of the five categories of fibers listed above are thought to make use of these neurotransmitters. Therefore, it has been suggested that TH-positive fibers in the cervical vagus could represent postganglionic sympathetic fibers, which are known to be noradrenergic (Lundberg et al., 1976; Kawagishi et al., 2008; Seki et al., 2014; Verlinden et al., 2016).

In this study, we want to provide further anatomical data on these catecholaminergic fibers in the human vagus nerve. Specifically, we have studied to what extent vagal fascicles change their fiber composition and what this means for the location of the vagal catecholaminergic fibers. We have ascertained the origin and direction of these catecholaminergic fibers and have determined their variability in absolute content between the left and right vagus nerve as well as along its cervical course and between subjects. These data may be very valuable for predictions and/or interpretations of the outcome of left or right vagus nerve stimulation and could be used for improvements and adaptations of the stimulation protocol.

## Materials and methods

### Human vagus nerves

Human cervical vagus nerves (*n* = 62) were dissected and collected from a total of 43 human cadavers in the dissection room of the Erasmus MC Rotterdam between October 2019 and December 2021. Cadavers had been standardly embalmed 24 to 72 h post-mortem with normal 4% formaldehyde fixation using the AnubiFiX prerinse method (Slieker et al., 2012; Theeuwes et al., 2020). Post-embalming time before extraction of the nerves varied between 3 and 54 months during which the cadavers were kept in containers with fenoxyethanol. Photographs of the nerves were taken *in situ* and after extraction. In 11 cases the superior cervical ganglion (SCG) and attached sympathetic trunk were collected together with the vagus nerve. After extraction, the nerves were stored in cold 4% paraformaldehyde in 0.05 M phosphate buffer (pH7.4).

### Immunohistochemistry

Selected pieces of the nerve were marked with ink and embedded sequentially together with either the left or right half of a mouse brain in 12% gelatin and 10% sucrose and hardened for 4 h in 10% formalin with 30% sucrose. Afterward, blocks of gelatin were stored overnight in 30% sucrose at 4°C. Gelatin blocks were frozen on a freezing microtome, sectioned transversally at 50 μm and collected sequentially in 0.1 M phosphate buffer in 8 or 16 numbered vials, such that every vial contained a complete 1 out of 8 (or 1 out of 16) series of sections of the whole gelatin block. Selected vials were prepared for free-floating immunohistochemical processing and mounted on glass slides where the individual vagus sections could be ordered based on their position within the block and the coronal level of the mouse brain.

Standard immunohistochemical protocols were used in order to differentiate between fibers of the vagus nerve. However, not all antibodies worked reliably in this embalmed material (see Table 1). As successful primary antibodies, we have used anti-myeline basic protein (MBP, Millipore), anti- tyrosine hydroxylase (TH, Abcam) and neurofilament, medium chain (NF, Sigma). No, or highly variable results were obtained with antibodies against choline acetyltransferase (ChAT, Millipore), calcitonin gene-related peptide (CGRP, Calbiochem), protein gene product 9.5 (PGP9.5, Enzo) and P2X purinoceptor 3 (P2X3, Neuromics). Prior to incubation sections’ endogenous peroxidase activity was blocked by rinsing them with 3% hydrogen peroxide. Antigenicity was improved by heating sections to 80°C. Sections were incubated overnight at room temperature with the primary antibody, free-floating in their vials under gentle agitation. Vials were subsequently rinsed three times in PB and could be processed for light microscopical staining using biotin-labeled secondary antibodies and avidin-biotin-peroxidase (ABC-kit, Vector) and diaminobenzidine (DAB) visualization of the antigen. For double or triple labeling purposes, selected vials were processed for immunofluorescence microscopy using appropriate fluorescent secondary antibodies for 2 h and counterstaining with DAPI (4′,6-diamidino-2-phenylindole). Sections were thoroughly rinsed and mounted on glass slides from a chrome alum solution and cover-slipped with Mowiol 4-88. DAB-stained sections were stored in the dark at room temperature for several months to years, whereas fluorescent sections were stored in the dark at 4°C for up to several weeks. Care was taken to simultaneously process left and right vagus nerve from the same body. In this way, variability due to different immunohistochemical runs were minimized.

**TABLE 1:** Primary antibodies used in this study.

Antibody	Abbreviation and company	Labels	Host	Dilution	Labeling
Myeline basic protein	MBP (Millipore)	axon myelination	chicken	1:1,000	+ ++
Tyrosine hydroxylase	TH (Millipore	catecholamine structures	mouse	1:500	+ ++
Neurofilament medium chain	NF (Sigma)	axons	mouse	1:1,000	+ +
Choline acetyl transferase	ChAT (Millipore)	cholinergic structures	goat	1:30,000	±
Calcitonin gene-related peptide	CGRP (Calbiochem)	nociception	goat	1:30,000	–
Protein gene product 9.5	PGP9,5 (Enzo)	pan neuronal marker	rabbit	1:10,000	–
P2X purinoceptor 3	P2X3 (Neuromics)	peripheral pain fibers	guinea pig	1:25,000	–

### Analysis

Sections prepared for light microscopy were examined and photographed with a Leica DMRB microscope. Selected series were scanned with a Hamamatsu NanoZoomer 2.0-RS. Fluorescent sections were imaged with a Zeiss Axio Imager 2 and, for confocal microscopy, a Zeiss LSM 510 or Leica stellaris 5 confocal microscope. For three nerves a three-dimensional reconstruction was made using either a 1 out of 4 or a 1 out of 8 series of serial sections. Individual vagus nerve sections were ordered with respect to the level of the mouse brain in the gelatin sections. Reconstructions and rotating video segment were prepared by manually entering fascicle contours in Neurolucida (MBF Bioscience).

In order to indicate and follow the position and density distribution of TH-labeled fibers within the vagus nerve, DAB-labeled sections scanned with the same Nanozoomer scanning settings, were entered in the Fiji software application that, after thresholding and density color-coding, allowed to calculate and visualize the relative density of TH-positive fibers at different levels of the vagus nerve.

For quantification of the TH-content of selected left and right vagus nerves of 20 bodies, approximately 5 mm of the cervical vagus nerve were extracted at four different cranio-caudal levels (see section “Results” and “Distribution and quantification of TH-positive fibers in the cervical vagus nerve”). Using cutouts of the nanozoomer images entered in the Fiji software package, individual fascicles were outlined and thresholded for TH-staining. In order to diminish the subjectivity of the thresholding process, the same TH-selection threshold was used for every section of the same pair of nerves and repeated for three sections at every level. This approach was considered to be the most reliable, because the left and right nerve of every pair simultaneously underwent an identical immunostaining procedure. In this way the total effective (i.e., summed) fascicle surface area of three sections of every vagus nerve level as well as the TH-surface per fascicle and summed per section were determined and averaged for the three vagus nerve sections taken at every level.

The fraction of the sympathetic fibers was calculated by dividing the TH-positive area by the effective surface area. For every slice the number of fascicles was counted. In order to examine the distribution of the TH-positive fibers amongst the fascicles, the standard deviation and variance between the TH-positive fractions of the fascicles from each nerve at every level were calculated. The average of the measurements from the three different sections of the same nerve at the same level were used for the statistical analyses performed with IBM SPSS Statistics (version 27). Differences between the left and right vagus nerves were tested for significance using the Student paired T-test when normality could be assumed (Shapiro–Wilk test > 0.05). Differences between the 4 levels were tested for significance using the Repeated Samples ANOVA Test, for which the data was tested for sphericity and normality. If the sphericity assumption was violated (Mauchly’s test of sphericity < 0.05), a Greenhouse-Geisser correction was used. *Post hoc* analyses were performed if a significant difference was found. Results were considered significant if *p* < 0.05.

## Results

### Organization of fascicles in the cervical vagus

Axons within peripheral nerves are bundled into a number of fascicles, each enwrapped by a sheet of connective tissue called perineurium. Fascicles are interconnected by the epifascicular epineurium that is formed by loose connective tissue. The nerve itself is surrounded by a dense layer of epineurium (Stewart, 2003). As the vagus nerve contains at least five different categories of fibers, it is relevant to know to what extent these fibers are already separated at the cervical level, which would imply that individual fascicles already contain specific and functionally related fibers. Conversely, it would be possible that at the cervical level no specific organization is present, or that the fascicles are still arranging themselves into functional entities. Therefore, we wanted to know if the fascicle structure of the cervical vagus resembles a cable-like structure, with no or only a few changes in fascicle composition throughout its length, or a plexiform structure, with ample changes in the configuration of fascicles (Sunderland, 1945; Jabaley et al., 1980; Stewart, 2003; Upadhye et al., 2022).

**FIGURE 1:**
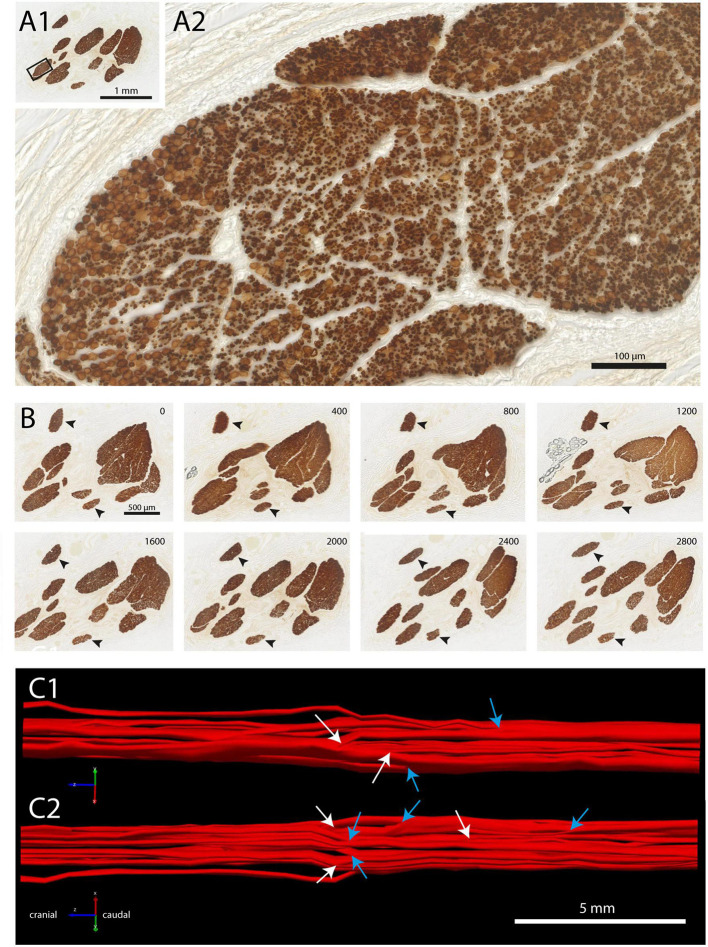
Fascicle composition of vagus nerve at mid-cervical level. **(A1,A2)** Staining with myeline basic protein (MBP) showing ten individual fascicles **(A1)**, inset is shown magnified in panel **(A2)**. Note that within this fascicle, several subgroups can be recognized. **(B)** Branching and joining of fascicles in a stretch of 2.8 mm of the cervical vagus. Only two fascicles (arrowheads) did not show any change in fiber composition. **(C1,C2)** Reconstruction of fascicle composition of 20 mm of the cervical vagus seen from medial **(C1)** and lateral **(C2)**. Cranial is to the left. White arrows depict branching of fascicles, blue arrows indicate joining of fascicles. A video showing a 3D-rotation of this segment of cervical vagus is found as Supplementary Video 1.

**FIGURE 2:**
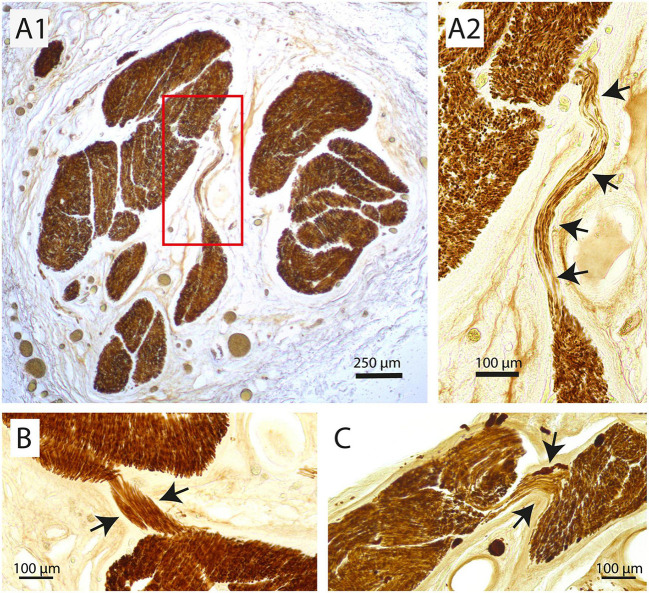
Examples of redistribution of small parts of a fascicle to another fascicle at mid-cervical level. **(A1,A2)** Here, several tens of myelinated fibers travel more than 500 μm in a transverse direction to join another large fascicle. **(A2)** Shows a magnification of the inset in panel **(A1)**. Arrows indicate strands of fibers that jump from one fascicle to another. **(B,C)** Two other examples where only a small part of a fascicle (arrows) detaches itself to join another fascicle at virtually the same cranio-caudal level of the vagus nerve.

Fascicle number and structure of the vagus nerve at mid-cervical level could be readily examined by staining for myelin basic protein (MBP). Examination of individual fascicles revealed that the fiber composition was wide-ranging and changed constantly (Figure 1). Moreover, fascicles often split and fused along their course (Figures 1B, C, also see Supplementary Video 1). Indeed, 3D-reconstructions of a 2 cm segment of the left cervical vagus, showed many of these split and fuse points (Figure 1C). Occasionally, small bundles dissociated themselves from a fascicle to take a virtually radial course to join another fascicle located either next to them or even on the other side of the nerve (Figure 2).

### TH-immunohistochemistry identifies noradrenergic fibers in the cervical vagus nerve

Various antibodies were used in order to examine the fiber content of the cervical vagus (Table 1). However, results were not always satisfactorily presumably due to suboptimal fixation and/or prolonged storage of the embalmed human cadavers. Apart from MBP also tyrosine hydroxylase (TH) and the medium-sized polypeptide neurofilament (NF) staining worked reliably in both DAB-processed sections for light microscopy as well as for fluorescence microscopy (Table 1).

**FIGURE 3:**
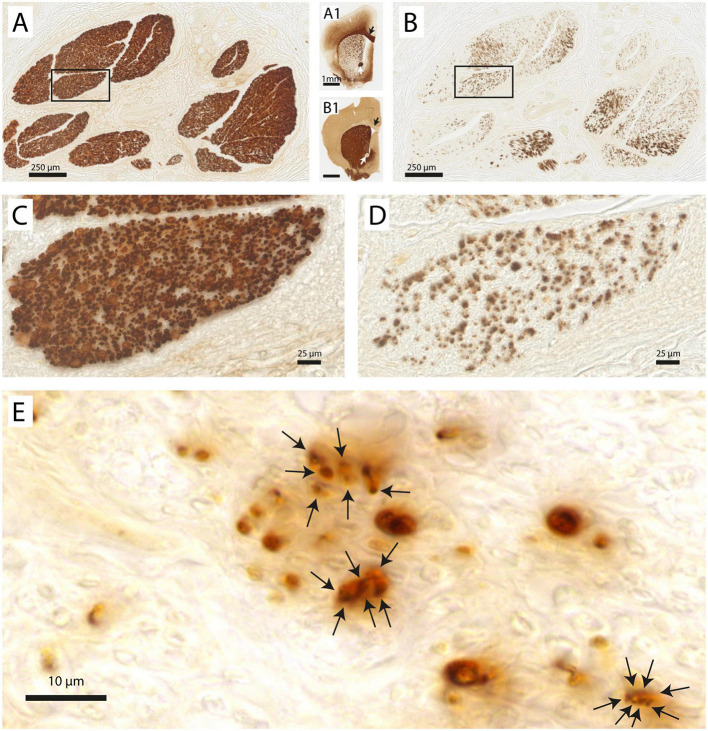
Vagus nerve fascicles contain TH-positive structures. **(A)** Vagal fascicles at mid-cervical level demonstrate evenly distributed MBP-labeling indicating a generally uniform density of myelinated fibers throughout all vagal fascicles. **(A1)** MBP-labeling of mouse forebrain embedded within the same gelatin block. Note the MBP-staining of the corpus callosum (black arrow), anterior commissure (white arrow) and internal capsule fascicles in the caudate-putamen. **(B)** Consecutive section stained for TH. Note distributed punctate labeling within most fascicles. **(B1)** TH-labeling of mouse forebrain. Note absence of labeling in the corpus callosum (black arrow) and anterior commissure (white arrow). Caudate-putamen now shows dense TH-labeling in its neuropil as dopaminergic terminals also are positive for TH. **(C,D)** Magnification of inset shown in panels **(A,B)**, respectively. **(E)** Detail of punctate TH-labeling. Larger punctae can be seen to be made up of several smaller ones with diameters of 1–2 μm.

**FIGURE 4:**
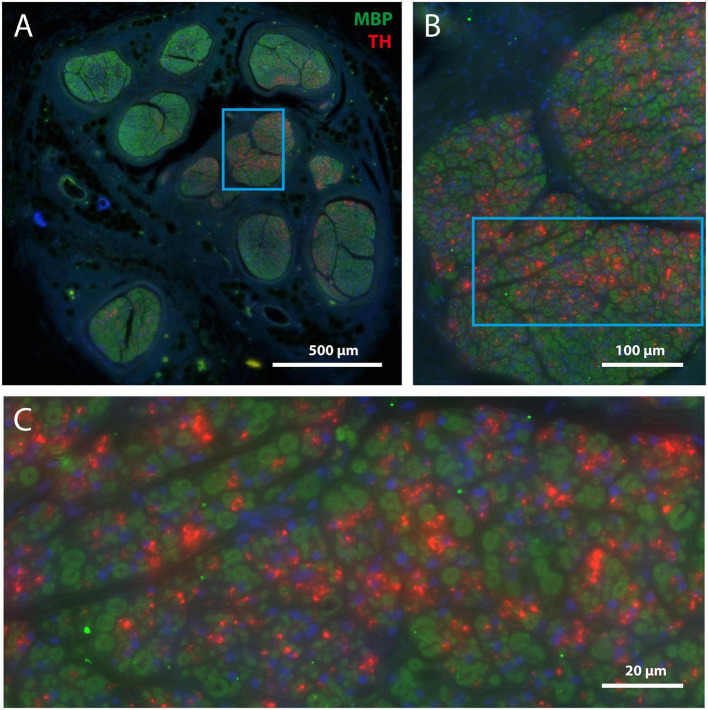
TH-positive structures are unmyelinated. **(A)** Overview of mid-cervical vagus nerve section double stained for MBP (green) and TH (red). **(B)** Magnification of framed area shown in panel **(A)**. **(C)** Magnification of framed area shown in panel **(B)**. Note that TH-positive structures are not surrounded by myeline (i.e., green) but are scattered between the myelinated fibers. Blue staining indicates DAPI, labeling cell nuclei.

As TH is an enzyme required for the production of the catecholaminergic neurotransmitters dopamine and (nor-) adrenaline, positive staining in the vagus nerve indicates the presence of dopaminergic and/or noradrenergic fibers. Indeed, Figure 3 shows two adjacent sections of the same vagus nerve fascicle stained for either MBP or NF. It can be observed that in the same region of the MBP profiles, heavy TH labeling is seen, usually presenting itself in small clusters made up of individual profiles in the submicron range. In order to see to what extent TH labeling is confined to the MBP profiles, double labeling with fluorescent microscopy was performed. From Figure 4, it can be appreciated that TH-profiles are actually fully separated from the MBP profiles as they are found completely in between and not within MBP profiles.

**FIGURE 5:**
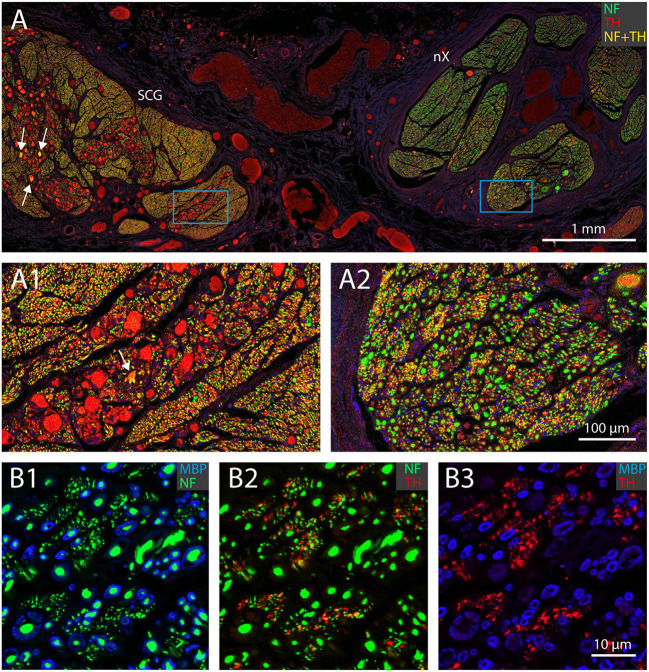
TH-positive structures represent unmyelinated nerve fibers. **(A)** Double fluorescent labeling of neurofilament (NF, green) and TH-positive structures (red) in a joint preparation of the superior cervical ganglion (SCG; left) and vagus nerve (nX; right). Note that all somata in the SCG are TH-positive, but only a few are also labeled for NF (yellow, arrows). **(A1,A2)** Magnification of framed areas in panel **(A)**. Virtually all fibers within the SCG are TH- and NF-positive (yellow), whereas in the vagus nerve many large profiles are NF positive, in selected areas small profiles are TH- and NF-positive. **(B1-B3)** Triple labeling of myeline (MBP, blue), neurofilament (NF, green) and tyrosine hydroxylase (TH, red) of part of a vagal fascicle. Note that the red TH profiles in panel **(B3)** match the location of the green NF profiles in panel **(B2)** (although they do not have the exact same shape) and represent unmyelinated fibers as shown in panel **(B1)**. All myelinated fibers are TH-negative **(**cf. **B1,B2)**.

In fact, as all TH-profiles are without myelination, we wondered if the TH-profiles really reflect axons and are not an artefact related to e.g., the embalming procedure. Double labeling with an antibody against neurofilament (NF), however, positively identified the TH-profiles as neuronal tissue (Figure 5). In addition, as can be seen in Figures 5A, A1, both fibers as well as ganglion cells in the superior cervical ganglion (SCG) both labeled intensely positive for TH, which is in agreement with the noradrenergic identity of postganglionic sympathetic elements. Note that most SCG neurons were NF-negative as only a few were double labeled (arrows in Figures 5A, A1). A reduction of NF-positive SCG neurons in the elderly was reported by Liutkiene et al. (2007). SCG Triple fluorescent labeling and confocal microscopy of the vagus nerve (Figure 5B) furthermore confirmed that most large caliber NF-positive fibers were myelinated and TH-negative, whereas individual as well as groups of TH-positive fibers colocalized with NF. Note that although the location of the TH-profiles is clearly covered by NF-profiles, the appearance of the former is clearly fuzzier compared to the punctate labeling of the NF. This suggests that the subcellular location of both types of molecules may be different, possibly due to issues with tissue preservation.

### Origin and direction of TH-positive fibers in the cervical vagus nerve

**FIGURE 6:**
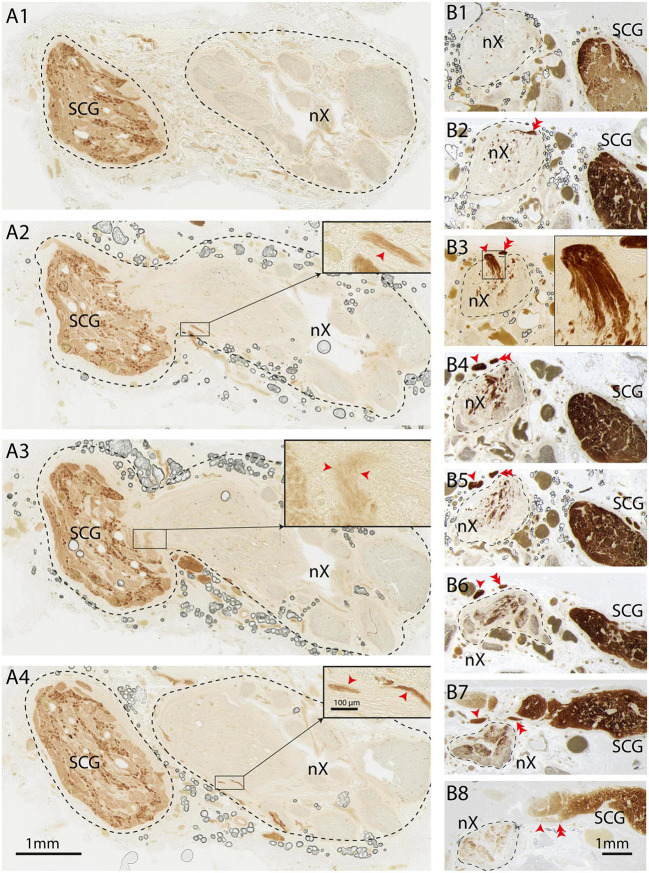
TH-positive fibers from the sympathetic trunk enter the vagus nerve and take a caudal course. “Direction” of fibers should be seen in relation to the normal flow of information (i.e., direction of actionpotentials) through the axons. **(A1–A4)** Series of sequential sections (distance between sections 600 μm) from cranial **(A1)** to caudal **(A4)**. SCG is shown on the left, vagus nerve on the right. In panels **(A2,A3)** the SCG is attached to the vagus nerve and small bundles of TH-fibers can be observed to enter the nerve. At **(A1)** (cranial) and **(A4)** (caudal-most level) the SCG is not connected to the vagus. Insets show details, red arrowheads indicate TH-fibers. Note that in panel **(A1)** no TH-fibers can be observed, which indicates that the TH-fibers upon entering the vagus nerve take a caudal route. **(B1–B8)** Series of sequential sections from cranial **(B1)** to caudal **(B8)**. The vagus nerve is demarcated by a striped line at the left side, whereas the SCG is positioned at the right side. In panel **(B8)** two fascicles detach from the sympathetic trunk and course in cranial direction as they can be followed from the section shown at **(B8)** to the level shown at **(B3)** (single arrowhead; inset in panel **(B3)** shows magnification of entrypoint). The other TH-positive fascicle can be seen to have entered the vagus nerve at the section shown in panel **(B2)** (double arrowhead) and **(B3)** Note that virtually no TH-fibers are present within the vagus nerve in the more cranial section shown in panel **(B1)** indicating that after entering the vagus nerve the TH-fibers course in caudal direction as can be appreciated by the TH-content within the vagus nerve in panels **(B4–B8)**. nX, vagus nerve; SCG, superior cervical ganglion.

These results clearly demonstrate that large quantities of TH-positive axons are present within the cervical vagus nerve. We wondered how and where these nerve fibers entered the vagus nerve. In principle any, or a combination, of three sources may be responsible. Firstly, these fibers may exit from the medulla together with the other constituents of the vagus nerve and as such could be efferent fibers originating in the medulla (i.e., within or in around the dorsal motor nucleus of the vagus or ambiguus nucleus) and traveling to their target organs. Another possibility would be that these fibers are afferent fibers and will terminate in the medulla (i.e., within or around the nucleus of solitary tract or spinal trigeminal nucleus). However, the sympathetic trunk, ascending close to the vagus nerve, could also be a likely candidate. Indeed, in a number of instances, during dissection of the vagus nerve, there were indications that the SCG and vagus nerve were directly connected (Figure 5A). In these cases, the SCG was dissected out with the vagus nerve, and both were subsequently processed simultaneously (e.g., Figure 5A). Indeed, when examining serial sections, we observed a case where the SCG became directly attached to the vagus nerve and allowing several thin bundles of fibers to enter the vagus nerve upon which a caudal route within the vagus nerve was taken by these fibers (Figure 6A). In another instance two TH-positive fiber bundles dissociated themselves from the sympathetic trunk close to the SCG, subsequently ascending parallel to vagus nerve for several mm and finally joining it. Here, their course was reversed such that the TH-labeled fibers followed a descending course (Figure 6B).

No examples were observed that showed that major quantities of TH-positive fibers coursed in ascending direction toward the brainstem. However, it should be noted that within the nodose ganglion a subpopulation of ganglion cells was TH-positive (Verlinden et al., 2016).

### Distribution and quantification of TH-positive fibers in the cervical vagus nerve

**FIGURE 7:**
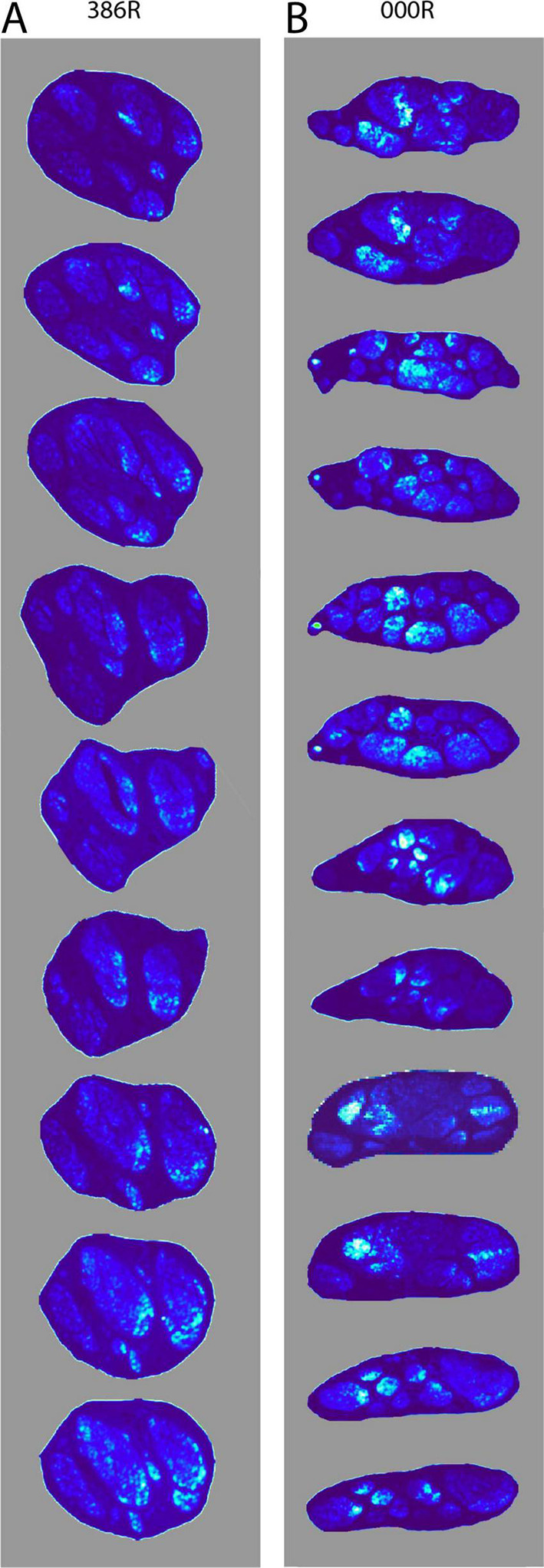
Density distribution of TH-positive fibers through sequential levels of the cervical vagus. **(A)** The density distribution is shown at nine levels, representing 45 mm shown from cranial (top) to caudal (bottom) of the right mid-cervical vagus (386R). Note that small fascicles present at the top distribute themselves throughout multiple fascicles. **(B)** Shows the density distribution at twelve levels, representing 90 mm of mid-cervical vagus 000R. Here too, the TH-positive fibers do not take up a fixed position, but may drift throughout the vagus, yet they tend to stay clustered.

From our observations, we surmised that most, if not all, TH-positive fibers entered the vagus as small fascicles that detached from the SCG and cervical sympathetic trunk. Upon entering the vagus, we wondered if these fascicles remained intact or, following the many ramifications of the vagal fascicles, became distributed throughout the vagus. As a first step we used the open-source software package Fiji (Schindelin et al., 2012) to define a threshold in order to localize TH-positive fibers and compared the intensity of staining as a measure of the surface density to the TH-positive fibers throughout the cervical part of the vagus nerve. Figure 7 shows that the distribution of TH-positive fibers is not fixed throughout the nerve but can shift throughout the various fascicles that make up the vagus at any cervical level.

**FIGURE 8:**
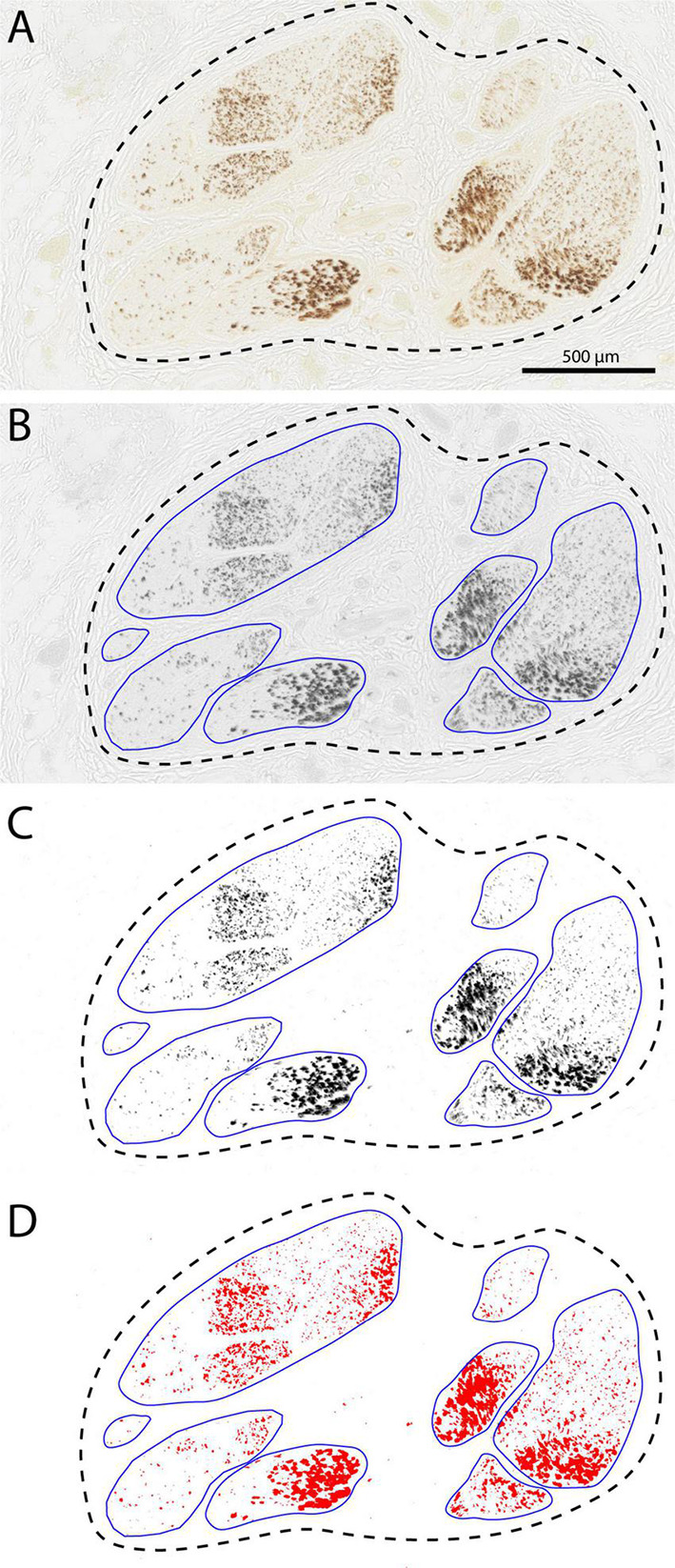
Determining surface area of the TH-labeled fibers using Fiji software. **(A)** Starting image captured with Nanozoomer (Hamamatsu). **(B)** Conversion to gray values using automatic adjustment for contast and brightness and manual delineation of the fascicles. **(C,D)** Thresholding and determination of the positive surface area within the individual fascicles and final calculation of summed fascicle area and percentage coverage by TH-labeling. Data for this particular section were: number of fascicles: 8, total fascicle surface area: 1.294 mm^2^. and average TH-coverage: 15.66%.

Figure 7 also indicates that the number of TH-positive fibers may not be constant throughout the different cervical vagus levels. However, due to their small diameter it proved impossible to quantify the absolute number of TH-fibers at any given level. In literature, a generally accepted method to determine the density of TH-fibers is to determine the area of labeled structures within a given surface. Here, again using the Fiji software package (Figure 8), we have determined the surface area after thresholding the TH-labeled fibers of all fascicles making up the vagus diameter at four different cervical levels, from cranial to caudal: level 1: SCG level; level 2: level of hyoid bone; level 3: cranial border of posterior aspect of cricoid cartilage; level 4: passage of omohyoid muscle. This will not only enable a comparison of the density of labeled TH-profiles throughout a single vagus nerve but also allows comparisons between the left and right vagus and between different individuals.

**FIGURE 9:**
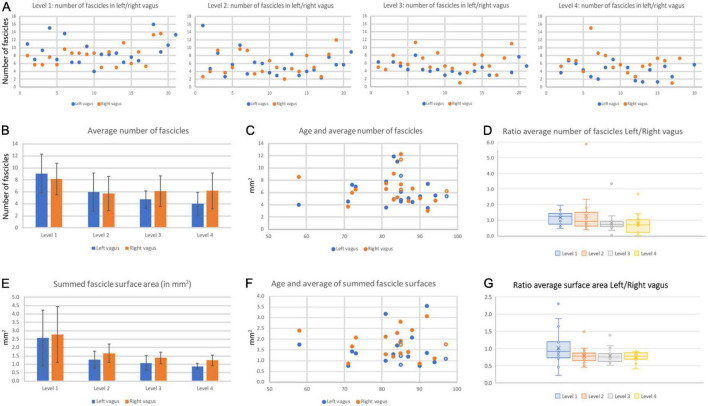
Fascicle number and surface area of left and right cervical vagus nerves. **(A)** Number of fascicles observed in the 20 pairs of examined vagus nerves at the four levels. On the x-axis the blue dots represent the left vagus nerve and the matching orange dots represent the right vagus nerve of all individual pairs. Note that in levels 3 and 4, generally more fascicles were found in right nerves. Also note that data were not always available for every level. **(B)** Average number of fascicles for all left and right nerves for every level. Bars represent standard deviation. **(C)** The number of fascicles per nerve was not correlated to the age of the specimens. **(D)** Box-whisker plot of the ratio left/right nerve fascicle number at the four levels. Note that at cranial-most levels the average ratio is close to 1.0 whereas at the caudal levels most ratio’s fall below 1.0, indicating there are more fascicles present in the right vagus nerve as compared to the left one. **(E)** Average surface area of the summed fascicle areas at the four examined levels. Note high variability at the cranial-most level. No significant statistical differences were noted between left and right vagus nerve, although paired analysis indicates that the right vagus nerve at caudal cervical levels has a significant larger surface area compared to the left vagus nerve. **(F)** Vagal surface area is not related to the age of the specimen. **(G)** Box-whisker plot of the ratio left/right nerve surface area at the four levels. Note that only level 1 measurements average around 1.0, whereas at the other levels the right vagus generally has a larger cross-sectional surface area compared to the left nerve. Open symbols represent data from female specimens.

For this part of the study twenty embalmed cadavers (16 male and 4 females; varying in age from 56 to 97 years, average 82: Table 2) were selected for extraction of the left and right cervical vagus nerves. The nerves were photographed *in situ* and the four levels were indicated with a marker at the surface of the vagus before extraction. In total 38 vagus nerves were harvested from the 20 bodies, as in two cases it was not possible to extract the right vagus nerve (Table 2). Approximately 5 mm of vagus was dissected at the four levels and processed for TH-immunohistochemistry, for some nerves, however, it was not possible to obtain material at levels 3 or 4 (Table 2). For all nerves and at all obtained levels, the number as well as the surface area of the individual fascicles was determined (Figure 9).

**TABLE 2:** Cross sectional surface area of examined left and right vagus nerves and their average TH-coverage.

				Left vagus nerve								Right vagus nerve							
				**Sum of cross-sectional fascicle surface areas (in mm^2^)**				**Average%TH-coverage**				**Sum of cross-sectional fascicle surface areas (in mm^2^)**				**Average%TH-coverage**			
**Specimen number**	**Age (years)**	**Embal-ming time (months)**	**M/F**	**Level 1**	**Level 2**	**level 3**	**level 4**	**Level 1**	**Level 2**	**level 3**	**level 4**	**Level 1**	**Level 2**	**level 3**	**level 4**	**Level 1**	**Level 2**	**level 3**	**level 4**
133	84	51	**M**	2.576	1.689	0.844	no data	0.709	0.422	1.852	no data	3.228	2.186	1.445	no data	1.209	0.832	0.987	no data
239	83	36	**M**	3.021	0.770	0.769	0.621	0.869	0.099	0.210	0.062	1.617	1.105	1.240	0.818	0.384	0.272	0.288	0.435
269	81	33	M	1.195	0.943	0.865	0.993	0.003	0.014	0.009	0.002	1.210	1.294	1.395	1.237	0.019	0.011	0.010	0.016
302	72	29	M	2.674	1.309	0.814	0.928	0.049	0.068	0.038	0.093	2.187	1.490	1.581	1.360	0.024	0.033	0.021	0.014
321	83	27	**M**	3.597	1.339	no data	no data	0.470	0.051	no data	no data	3.201	0.962	0.736	0.551	0.884	0.020	0.097	0.064
344	90	26	M	0.807	0.734	0.772	0.673	16.199	23.365	26.790	14.478	0.814	0.888	0.888	0.887	15.206	29.640	21.045	17.834
386	85	20	M	2.212	1.486	1.948	no data	0.396	1.439	0.453	no data	4.363	2.233	1.808	no data	0.883	1.036	0.131	no data
401	85	18	**F**	1.525	1.550	0.983	0.857	7.011	11.730	2.781	4.550	2.157	1.980	1.325	1.526	21.788	23.728	27.941	30.809
407	58	18	M	3.946	0.989	0.943	1.147	2.677	2.848	2.042	1.054	5.354	1.400	1.330	1.523	4.586	6.694	5.674	17.116
426	97	17	**F**	1.349	0.905	1.032	1.067	6.525	11.418	7.605	9.172	2.984	1.560	1.306	1.225	8.145	16.628	20.749	21.310
444	73	15	M	2.335	1.148	1.089	0.787	1.867	3.051	2.823	2.767	2.234	2.280	1.853	1.908	0.876	0.655	2.456	2.382
452	81	14	M	5.029	2.671	1.808	no data	7.300	4.454	1.888	no data	3.061	1.793	2.126	1.438	2.040	6.884	11.391	5.530
463	87	13	M	1.325	1.427	1.020	0.949	19.941	27.177	16.255	19.133	1.455	1.641	1.427	1.041	16.403	23.747	26.238	27.216
465	71	15	M	0.743	0.833	0.703	0.724	4.878	4.154	1.460	1.639	0.902	0.828	0.858	0.846	3.187	1.274	1.551	4.847
471	94	12	M	1.178	1.007	0.768	0.769	13.388	17.036	21.979	13.671	1.342	1.237	0.957	0.871	3.814	10.739	8.571	9.941
477	88	13	M	4.682	1.323	1.109	1.186	6.983	8.559	11.019	11.513	3.960	2.890	1.560	1.299	4.292	4.496	3.450	3.950
504	92	8	M	3.324	0.703	0.682	0.737	5.448	9.322	23.977	0.917	no data	no data	no data	no data	no data	no data	no data	no data
522	85	7	M	4.370	1.110	1.069	0.885	0.782	3.102	3.888	2.893	1.903	1.341	1.056	1.045	5.529	8.396	7.852	8.695
538	85	4	**F**	0.935	0.797	0.821	0.747	3.050	6.072	3.757	3.955	4.199	1.364	1.073	1.087	2.532	1.106	1.115	1.072
540	92	3	M	6.358	2.093	2.145	no data	2.458	0.065	0.085	no data	6.898	2.202	1.544	1.595	3.418	7.985	9.785	4.108
Mean	83.3	19.0		2.659	1.241	1.062	0.871	5.050	6.722	6.785	5.727	2.793	1.614	1.343	1.192	5.012	7.588	7.861	9.138
SD	9.1	11.8		1.605	0.495	0.427	0.172	5.642	7.955	8.829	6.236	1.613	0.556	0.364	0.346	6.169	9.295	9.381	10.011

The number of fascicles varied considerably between different levels of the same nerve, between the left and right nerve and between individual nerves (range for level 1: 4–16 and for level 4: 1–16, Figure 9A). A repeated measures ANOVA showed that the average number of fascicles in both the left and right vagus nerves differed significantly between the 4 levels [left vagus: *F*(3,42) = 19.388, *P* < 0.001; right vagus: *F*(3,48) = 5,27, *P* = 0.003]. *Post hoc* analysis (data not included) indicated that for both sides significant changes were only found between level 1 and the other levels. The most cranial level averaged 9,06 (left vagus) to 8,12 (right vagus) fascicles; at the most caudal level the number of fascicles shrank by more than 50% for the left vagus (4,04 fascicles), whereas in the right vagus the average number of fascicles diminished by less than 20% (to 6,28 fascicles). Indeed, for levels 3 and 4 of Figure 9A, it can be observed that the right vagus usually contained more fascicles than the left one. No obvious correlation could be found with respect to the age or gender of the cadavers from which the nerves were taken (Figures 9C, D). The total fascicle surface area, i.e., the effective area containing nerve fibers, averaged around 2.5 mm^2^ but diminished to about 1 mm^2^ at level 4 (Figure 9E), indicating that a major part of the vagus nerve fibers detached from the main nerve between levels 1 and 4. Although at level 1 no difference was noted, at levels 2–4, Student paired T-tests indicated that the right vagus nerve of a pair generally had a larger effective surface area as compared to left nerve (level 1–4: *p* = 0.550, *p* = 0.004, *p* < 0.001, *p* < 0.001, respectively). This can also be seen in Figure 9G where the ratio of left to right vagal surfaces averaged below 1 for levels 2–4.

**FIGURE 10:**
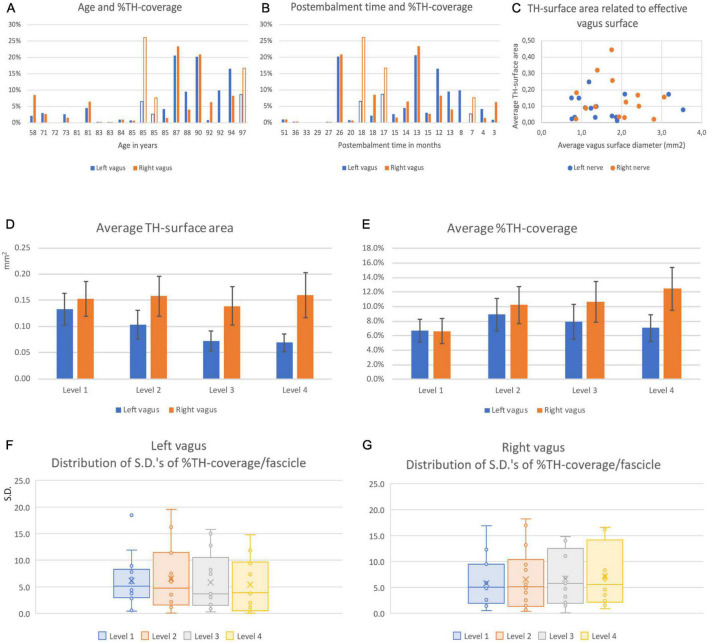
Coverage and distribution of TH-positive fibers in left and right vagus nerves. **(A)** Average surface percentage of total fascicle area ( = %TH-coverage) of the left (blue) and right (orange) vagus nerve related to the age of the specimens. Note large differences between individuals and between individual left and right nerves. **(B)** % TH-coverage related to post-embalming period. Note that the longest post-embalming periods (51–27 months) demonstrated the least coverage. **(C)** Relation between the averaged and summed surface areas of the vagus nerve and of the averaged and summed surface areas of the TH-compartments. **(D)** Average TH-surface area at the four examined cervical levels. Note that the surface generally diminished for left vagus nerves but remained at the same overall value for the right vagus nerve. **(E)** TH-surface area as related to the total effective nerve surface area of the summed fascicle surfaces. From panels **(C,D)** it can be surmised that the TH-coverage of the left nerve kept pace with the diminishing surface of the nerve whereas this increased for the right nerve. **(F,G)** Box-whisker plots of the standard deviations of the covering percentage of the individual fascicles determined at the four cervical levels for the left **(E)** and right **(F)** nerves. No significant differences were found indicating that the differences in TH-coverage noted between individual fascicles (e.g., see Figure 8) did not diminish at more caudal cervical levels of both nerves. Filled en open bars in panels **(A,B)** denote male and female data.

Subsequently, for every fascicle the area covered by TH-fibers was determined as shown in Figure 8. This was used to calculate the total TH-area in mm^2^ and as a percentage of the total fascicle area at all levels (Figure 10). It appeared that the percentage of TH-coverage averaged over the four levels varied considerably (range: 0.01–26,07%). As we were concerned that this could be due to the age of the cadavers from which the specimens were taken or to the length of the embalming period, we correlated these two variables with the TH-coverage (Figures 10A, B). Although the highest TH-coverages were observed nerves of the eldest specimens, no significant correlation between age and TH-content was found (paired samples correlation 0.403, *p* = 0.153, Figure 10A). However, Figure 10B indicates that the five cases with the longest embalming period (>26 months) all showed virtually no TH-labeling in contrast to the less long stored cases. Therefore, it was decided to exclude these five cases from further analysis. For the remaining cases no relation was found between the size of the vagus nerve and the number of TH-fibers (Figure 10C).

A Student paired *T*-test indicated no significant difference for the TH-positive fractions between the left (mean = 7.35%, SD = 6.94%) and right (mean = 9.62%, SD = 8.58%) vagus nerve (*p* = 0.228). For the left vagus nerve, a repeated measures ANOVA with Greenhouse-Geisser correction showed no significant difference between the 4 levels [*F*(1.482,16.300) = 2.893, *p* = 0.096]. A repeated measure ANOVA for the right vagus nerve indicated a significant difference between the 4 levels [*F*(3,36) = 5.950, *p* = 0.002]. *Post hoc* analysis showed that the TH-percentages in level 1 were significantly lower compared to levels 2, 3 and 4 [MD = −3.858 (95%CI, −6.645 to −1.071), *p* = 0.011; MD = −4.308 (95% CI, −7.074 to −1.542)%, *p* = 0.005; MD = −4.846 (95% CI, −7.856 to −1.836)%, *p* = 0.004, respectively].

Finally, we wanted to know if the distribution of TH-positive fibers within individual fascicles changed when moving in a caudal direction. Therefore, we compared the standard deviations (S.D.) of the average coverage between individual fascicles from cranial to caudal (Figures 10F, G). As a repeated measures ANOVA with Greenhouse-Geisser correction showed no significant difference between the S.D.’s of TH-percentages in fascicles between different levels (Left: *F* = 2.109, *p* = 0.148; right: *F* = 0.424, *p* = 0.737), no indication was found that the TH-fibers distributed themselves more equally over the fascicles making up the vagus nerves.

## Discussion

The present study confirms earlier reports that the cervical part of the human vagus nerve harbors a variable but often considerable number of TH-positive structures (Seki et al., 2014; Verlinden et al., 2016). We demonstrate that these TH-positive structures can be considered as unmyelinated nerve fibers as they have no MBP coating but do contain neurofilament. Therefore, the TH-positive fibers cannot be attributed to artefactual staining due to e.g., the embalming process, although, in contrast, we did note that prolonged embalming may considerably diminish TH-antigenicity. Furthermore, we show that at least a major component of these TH-fibers is derived from the SCG as has been suggested by others (Muryobayashi et al., 1968; Seki et al., 2014; Verlinden et al., 2016; Mitsuoka et al., 2017). In addition, we have studied the number and distribution of the TH-fibers at different cervical levels and conclude that the TH-content of the left vagus diminishes in caudal direction, whereas the TH-content of the right vagus is retained at all investigated levels. TH-fibers do not take up a specific position within the cervical vagus but may drift through different fascicles in a clustered manner. Although not specifically investigated and numbers were low, no indications were found that the vagal TH-content relates to either age or sex (Seki et al., 2014). A comparison between the results of different studies is shown in Table 3.

**TABLE 3:** Comparison of data obtained in different studies on human vagus nerves.

	Number of fascicles		Summed surface area (mm2)		TH-coverage (%)	
	**Left vagus**	**Right vagus**	**Left vagus**	**Right vagus**	**Left vagus**	**Right vagus**
References*	**6.1 (*n* = 20)**	**6.6 (*n* = 19)**	**1.58 (*n* = 20)**	**1.77 (*n* = 19)**	**7.51 (*n* = 15)**	**9.63 (*n* = 14)**
Seki et al., 2014	6.5 (*n* = 28)	9.1 (*n* = 29)	1.32 (*n* = 28)	1.84 (*n* = 29)	3.97 (*n* = 28)	5.47 (*n* = 29)
Verlinden et al., 2016	5 (*n* = 11)	8 (*n* = 11)	0.75 (*n* = 11)	1.09 (*n* = 11)	1.9 (*n* = 11)	3.3 (*n* = 11)
Upadhye et al., 2022 **	6.6 (*n* = 8)		1.32 (*n* = 8)			
Hammer et al., 2018 **	5.2 (*n* = 51)					

*Data represent averages of levels 1, 2, 3, and 4, other data are from the level used for cervical vagus nerve stimulation, which would correspond to mid-cervical level. **No difference between left and right vagus nerve was made in these studies.

### Origin and course of TH-fibers in the cervical vagus nerve

Tyrosine hydroxylase is the rate-limiting enzyme for the production of the catecholamines dopamine and (nor-) adrenaline (Nagatsu et al., 1964). TH-positive fibers in the cervical vagus are also positive for dopamine beta-hydroxylase (Verlinden et al., 2016), which converts dopamine into noradrenaline. Therefore, it is highly likely that the vagal TH-fibers represent noradrenergic nerve fibers. As postganglionic sympathetic fibers are known to be noradrenergic, it is generally assumed that these vagal catecholaminergic and TH-positive fibers represent sympathetic fibers (Muryobayashi et al., 1968; Lundberg et al., 1976; Kawagishi et al., 2008; Onkka et al., 2013; Seki et al., 2014; Verlinden et al., 2016). Indeed, in our study, we have found several instances where thin fascicles of TH-positive fibers connect the SCG with the vagus. Upon entering the vagus nerve these fibers do not continue in cranial direction but follow a descending, i.e., caudal route. This agrees well with observations of Verlinden et al. (2016), who did not observe TH-positive fibers in the intracranial course of the vagus. These results taken together make it highly likely that the TH-positive fibers in the cervical vagus are derived from the sympathetic trunk at the level of the SCG and represent postganglionic noradrenergic sympathetic fibers that seem to course in a descending direction. In theory, the TH-fibers could also originate as postganglionic fibers from lower levels and be derived from e.g., the coeliac or stellate ganglion connections with the vagus nerve and, ascending in the vagus, re-enter the sympathetic trunk to be distributed to their targets. However, in animal studies, several reports found evidence that catecholaminergic fibers in the vagus nerve originate from ganglion cells located in the SCG (Muryobayashi et al., 1968; Liedberg et al., 1973; Ahlman et al., 1978).

It is remarkable that our study, in agreement with earlier studies (Seki et al., 2014; Verlinden et al., 2016), noted a high variability in the presence of TH-positive fibers, both between cadavers as well as inter-individually between the left and right vagus nerve. This was not related to the variability in vagal diameter (determined as summed cross-sectional surfaces of fascicles). As an alternative, it might be due to chance, related to individual variations in the position of the SCG with respect to the vagus nerve. Indeed, upon dissection, we noted that sometimes the vagus nerve seemed to be completely unconnected to the SCG whereas at other times it seemed to be firmly attached, thus enabling easy transfer of fibers from trunk to vagus as shown in Figure 6A. However, thin strands of fibers emanating from the sympathetic trunk or SCG also could be followed in serial microscopical sections to enter the vagus nerve. These thin connections have also been described in macroscopical human cadaveric dissections (Mitsuoka et al., 2017).

Upon entering the vagus nerve, the TH-fibers distribute themselves over a selection of fascicles. In agreement with a recent study by Upadhye et al. (2022), we show that the fascicles themselves often split and merge along their course. In doing so, we note that clusters of TH-fibers can move from one fascicle to another, thereby redistributing themselves throughout the vagus nerve. However, as the standard deviation of the TH-coverage per fascicle did not change between levels, it can be stated that the redistribution does not lead to a completely random distribution throughout all fascicles. Therefore, although the cervical vagus nerve displays a highly plexiform arrangement, we propose that a certain degree of functional organization of specific fibers may still be present.

### Differences between left and right vagus nerve

It has been speculated what the target of the postganglionic sympathetic fibers in the cervical vagus might be (Muryobayashi et al., 1968; Seki et al., 2014; Verlinden et al., 2016). A sizeable proportion of TH-fibers has also been noted within the thoracic and abdominal parts of the vagus (Muryobayashi et al., 1968; Liedberg et al., 1973; Seki et al., 2014), suggesting that at least some of these fibers follow preganglionic parasympathetic fibers to their target organs. However, some TH-fibers may also follow branches of the vagus nerve that do not necessarily have a parasympathetic target. Indeed, we noted that at the caudal cervical levels of especially the left vagus nerve, coverage by TH-fibers was decidedly less compared to the higher cervical levels, which suggests that a major proportion of the TH-fibers of the left vagus nerve branched off. This coincided with a notably thinner left vagus nerve at lower cervical levels compared to higher regions (Figure 9A and Table 2). This is in contrast to the right vagus nerve, where a diminished size of the vagus did not appear to affect the absolute number of TH-fibers and as a consequence resulted in an increased ratio of TH-content at lower cervical levels (cf. Figures 10D, E). This could imply that branches of the left cervical vagus contain more TH-fibers than the branches coming from the right vagus. Cervical vagal branches would be expected to participate in innervation of the pharynx, superior laryngeal nerve and contributes to autonomous innervation of the heart by its superior cervical cardiac branch(-es). Although it is well-known that the innervation of the heart by the vagus is asymmetric as the right vagus nerve predominantly subserves the atria, including the sinu-atrial node, whereas the left vagus is thought to be more involved in the innervation of the ventricles and the atrioventricular node (Zandstra et al., 2021), it is less clear to what extent cervical branches are involved. Presently, we are not aware of any other systematic differences in the targets of the cervical branches from the left and right vagus nerve (Mitsuoka et al., 2017).

### Variation in TH-fiber content

From our study and that of others (Seki et al., 2014; Verlinden et al., 2016), we think it is now well established that the vagus nerve can harbor a significant number of sympathetic fibers that may use the vagus as a transport line to get to their target area. However, as only few or no TH-fibers were observed to move in the cranial direction upon entering the vagus nerve and no TH-fibers were found in the intracranial part of the vagus (Verlinden et al., 2016), we can state that the TH-fibers are not an original part of the vagus in the sense that their parent soma is located in the medulla or that their synaptic terminals end there. Rather, the TH-positive fibers use the course of the vagus nerve as an alternative route to reach their targets. The large variability in number of TH-fibers between subjects and even between the left and right nerve would suggest that the sympathetic use of the vagus nerve might be a chance process that is decided upon by developmental issues. In this respect little information is available suggesting to what extent or how the development of the sympathetic and parasympathetic pathways is linked (Schneider et al., 2009; Karemaker, 2017).

### Clinical implications

Vagus nerve stimulation is being used for many different indications. As the vagus nerve contains different types of somatic and visceral afferent and efferent fibers and considering that the efferent as well as the afferent targets might be somewhat different for the left and right vagus nerves, it is important to assess and understand the choice of side and direction of the vagus stimulation (Garamendi-Ruiz and Gomez-Esteban, 2019; Ahmed et al., 2020; Chang et al., 2020). We show now that an additional fiber type should be involved in assessing the effect of vagus nerve stimulation. As the participation of postganglionic sympathetic fibers in the vagus nerve varies considerably between subjects, while the course and targets between left and right nerve may be different, this assessment will not be straightforward. Indeed, as the TH-fibers, at least to some extent deriving from SCG neurons, but potentially also may originate at lower levels, their axons may collateralize to targets that are also normally supplied by the sympathetic system. This suggests that both “up” and “down” vagus stimulation using anodal block (Ahmed et al., 2020) may result in activation of variable sets of sympathetic effects. As such it may prove rather difficult to set stimulation parameters in such a way to produce reliable positive outcomes for the great variety of the clinical applications (Karemaker, 2022). Therefore, presently, clinicians should be aware that presently-used vagus nerve stimulation protocols may activate a rather variable population of TH-positive, and therefore most likely noradrenergic postganglionic sympaphetic, fibers. Although stimulation protocols are aimed to activate only myelinated fibers, it should be recognized that most afferent, i.e., viscerosensory, fibers ascending through the vagus nerve, are, like the TH-fibers, unmyelinated (Kupari et al., 2019). Indeed, their contribution to induce a successful therapy is still uncertain (e.g., Berthoud and Neuhuber, 2000; Chang et al., 2020; Cooper et al., 2021). Furthermore, animal research on the effects of vagal nerve stimulation often makes use of vagosympathetic trunk stimulation (Ardell et al., 2015). Hence, we propose that it will be important to assess if stimulation protocols aimed to stimulate mostly (or only) myelinated fibers without activating c-fibers also fail to activate the efferent unmyelinated TH-fibers. However, although unlikely, a potential positive contribution of stimulation of the TH-fibers in cervical vagus stimulation therapy should also be investigated.

## Data availability statement

The raw data supporting the conclusions of this article will be made available by the authors, without undue reservation.

## Ethics statement

Ethical review and approval was not required for the study on human participants in accordance with the local legislation and institutional requirements. Written informed consent for participation was not required for this study in accordance with the national legislation and the institutional requirements.

## Author contributions

JS, AV, and TR conceptualized this study. AV and JS provided the additional funding. LO, SM, and CK carried out the experiments and analyzed the material together with TR and JS. TR wrote the manuscript and designed the figures. All authors provided edits and approved the submitted version of the manuscript.

## References

[B1] Ahlman B. H. Lundberg J. M. Dahlstrom A. Larsson I. Pettersson G. Kewenter J. (1978). Evidence for innervation of the small intestine from the cervical sympathetic ganglia. *J. Surg. Res.* 24 142–149. 10.1016/0022-4804(78)90166-x 633879

[B2] Ahmed U. Chang Y. C. Cracchiolo M. Lopez M. F. Tomaio J. N. Datta-Chaudhuri T. (2020). Anodal block permits directional vagus nerve stimulation. *Sci. Rep.* 10:9221. 10.1038/s41598-020-66332-y 32513973 PMC7280203

[B3] Alvarez M. R. Alarcon J. M. Roman C. A. Lazaro D. Bobrowski-Khoury N. Baena-Caldas G. P. (2022). Can a basic solution activate the inflammatory reflex? A review of potential mechanisms, opportunities, and challenges. *Pharmacol. Res.* 187:106525. 10.1016/j.phrs.2022.106525 36441036

[B4] Ardell J. L. Rajendran P. S. Nier H. A. Kenknight B. H. Armour J. A. (2015). Central-peripheral neural network interactions evoked by vagus nerve stimulation: Functional consequences on control of cardiac function. *Am. J. Physiol. Heart Circ. Physiol.* 309 H1740–H1752. 10.1152/ajpheart.00557.2015 26371171 PMC4666982

[B5] Berthoud H. R. Neuhuber W. L. (2000). Functional and chemical anatomy of the afferent vagal system. *Auton. Neurosci*. 85, 1–17. 10.1016/S1566-0702(00)00215-0 11189015

[B6] Chang Y. C. Cracchiolo M. Ahmed U. Mughrabi I. Gabalski A. Daytz A. (2020). Quantitative estimation of nerve fiber engagement by vagus nerve stimulation using physiological markers. *Brain Stimul.* 13 1617–1630. 10.1016/j.brs.2020.09.002 32956868

[B7] Cooper C. M. Farrand A. Q. Andresen M. C. Beaumont E. (2021). Vagus nerve stimulation activates nucleus of solitary tract neurons via supramedullary pathways. *J. Physiol*. 599, 5261–5279. 10.1113/JP282064 34676533 PMC11328930

[B8] Garamendi-Ruiz I. Gomez-Esteban J. C. (2019). Cardiovascular autonomic effects of vagus nerve stimulation. *Clin. Auton. Res.* 29 183–194. 10.1007/s10286-017-0477-8 29071466

[B9] Hammer N. N. Loffler S. Cakmak Y. O. Ondruschka B. Planitzer U. Schultz M. (2018). Cervical vagus nerve morphometry and vascularity in the context of nerve stimulation - A cadaveric study. *Sci. Rep*. 8:7997. 10.1038/s41598-018-26135-8 PMC5964190 29789596

[B10] Jabaley M. E. Wallace W. H. Heckler F. R. (1980). Internal topography of major nerves of the forearm and hand: A current view. *J. Hand Surg. Am* 5 1–18. 10.1016/s0363-5023(80)80035-9 7365209

[B11] Karemaker J. M. (2017). An introduction into autonomic nervous function. *Physiol. Meas.* 38 R89–R118. 10.1088/1361-6579/aa6782 28304283

[B12] Karemaker J. M. (2022). The multibranched nerve: Vagal function beyond heart rate variability. *Biol. Psychol.* 172:108378. 10.1016/j.biopsycho.2022.108378 35688294

[B13] Kawagishi K. Fukushima N. Yokouchi K. Sumitomo N. Kakegawa A. Moriizumi T. (2008). Tyrosine hydroxylase-immunoreactive fibers in the human vagus nerve. *J. Clin. Neurosci.* 15 1023–1026. 10.1016/j.jocn.2007.08.032 18617399

[B14] Konstam M. A. Mann D. L. Udelson J. J. E. Ardell J. L. De Ferrari G. M. Cowie M. R. (2022). Advances in our clinical understanding of autonomic regulation therapy using vagal nerve stimulation in patients living with heart failure. *Front. Physiol.* 13:857538. 10.3389/fphys.2022.857538 35530511 PMC9068946

[B15] Kupari J. Haring M. Agirre E. Castelo-Branco G. Ernfors P. (2019). An atlas of vagal sensory neurons and their molecular specialization. *Cell Rep*. 27, 2508–2523.e4. 10.1016/j.celrep.2019.04.096 31116992 PMC6533201

[B16] Liedberg G. Nielsen K. C. Owman C. Sjoberg N. O. (1973). Adrenergic contribution to the abdominal vagus nerves in the cat. *Scand. J. Gastroenterol.* 8 177–180. 4121352

[B17] Liutkiene G. Stropus R. Pilmane M. Dabuzinskiene A. (2007). Age-related structural and neurochemical changes of the human superior cervical ganglion. *Ann. Anat*. 189, 499–509. 10.1016/j.aanat.2007.01.010 17910404

[B18] Lundberg J. Ahlman H. Dahlstrom A. Kewenter J. (1976). Catecholamine-containing nerve fibres in the human abdominal vagus. *Gastroenterology* 70 472–474. 1248708

[B19] Mitsuoka K. Kikutani T. Sato I. (2017). Morphological relationship between the superior cervical ganglion and cervical nerves in Japanese cadaver donors. *Brain Behav.* 7:e00619. 10.1002/brb3.619 28239529 PMC5318372

[B20] Muryobayashi T. Mori J. Fujiwara M. Shimamoto K. (1968). Fluorescence histochemical demonstration of adrenergic nerve fibers in the vagus nerve of cats and dogs. *Jpn. J. Pharmacol.* 18 285–293. 10.1254/jjp.18.285 5304397

[B21] Muthiah N. Joseph B. Varga G. Vodovotz L. Sharma N. Abel T. J. (2023). Investigation of the effectiveness of vagus nerve stimulation for pediatric drug-resistant epilepsies secondary to nonaccidental trauma. *Childs Nerv. Syst.* 39 1201–1206. 10.1007/s00381-022-05817-9 36602582

[B22] Nagatsu T. Levitt M. Udenfriend S. (1964). Tyrosine hydroxylase. The initial step in norepinephrine biosynthesis. *J. Biol. Chem.* 239 2910–2917. 14216443

[B23] Onkka P. Maskoun W. Rhee K. S. Hellyer J. Patel J. Tan J. (2013). Sympathetic nerve fibers and ganglia in canine cervical vagus nerves: Localization and quantitation. *Heart Rhythm* 10 585–591. 10.1016/j.hrthm.2012.12.015 23246597 PMC3758134

[B24] Payne S. C. Furness J. B. Stebbing M. J. (2019). Bioelectric neuromodulation for gastrointestinal disorders: Effectiveness and mechanisms. *Nat. Rev. Gastroenterol. Hepatol.* 16 89–105. 10.1038/s41575-018-0078-6 30390018

[B25] Saper C. B. Lumsden A. G. S. Richerson G. B. (2013). “The sensory, motor, and reflex functions of the brain stem,” in *Principles of neural science*, 5th Edn, eds Kandel E. R. Schwartz J. H. Jessel T. M. Siegelbaum S. A. Hudspeth A. J. (New York, NY: McGraw-Hill).

[B26] Schindelin J. Arganda-Carreras I. Frise E. Kaynig V. Longair M. Pietzsch T. (2012). Fiji: An open-source platform for biological-image analysis. *Nat. Methods* 9 676–682. 10.1038/nmeth.2019 22743772 PMC3855844

[B27] Schneider U. Schleussner E. Fiedler A. Jaekel S. Liehr M. Haueisen J. (2009). Fetal heart rate variability reveals differential dynamics in the intrauterine development of the sympathetic and parasympathetic branches of the autonomic nervous system. *Physiol. Meas.* 30 215–226. 10.1088/0967-3334/30/2/008 19179746

[B28] Seki A. Green H. R. Lee T. D. Hong L. Tan J. Vinters H. V. (2014). Sympathetic nerve fibers in human cervical and thoracic vagus nerves. *Heart Rhythm* 11 1411–1417. 10.1016/j.hrthm.2014.04.032 24768897 PMC4108556

[B29] Slieker J. C. Theeuwes H. P. Van Rooijen G. L. Lange J. F. Kleinrensink G. J. (2012). Training in laparoscopic colorectal surgery: A new educational model using specially embalmed human anatomical specimen. *Surg. Endosc.* 26 2189–2194. 10.1007/s00464-012-2158-y 22286275 PMC3392504

[B30] Stewart J. D. (2003). Peripheral nerve fascicles: Anatomy and clinical relevance. *Muscle Nerve* 28 525–541. 10.1002/mus.10454 14571454

[B31] Straube A. Eren O. (2021). tVNS in the management of headache and pain. *Auton Neurosci.* 236:102875. 10.1016/j.autneu.2021.102875 34500261

[B32] Sunderland S. (1945). Blood supply of the sciatic nerve and its popliteal divisions in man. *Arch. Neurol. Psychiatry* 54 283–289. 10.1001/archneurpsyc.1945.02300100057006 21006511

[B33] Theeuwes H. P. Potters J. W. Bessems J. Kerver A. J. Kleinrensink G. J. (2020). Use of the humeral head as a reference point to prevent axillary nerve damage during proximal fixation of humeral fractures: An anatomical and radiographic study. *Strategies Trauma Limb Reconstr.* 15 63–68. 10.5005/jp-journals-10080-1460 33505520 PMC7801902

[B34] Theiss P. Slavin K. V. (2022). Vagal nerve stimulation for treatment-resistant depression: An update on mechanism of action and clinical use. *Prog. Brain Res.* 270 97–104. 35396032 10.1016/bs.pbr.2022.01.001

[B35] Upadhye A. R. Kolluru C. Druschel L. Al Lababidi L. Ahmad S. S. Menendez D. M. (2022). Fascicles split or merge every approximately 560 microns within the human cervical vagus nerve. *J. Neural Eng.* 19: 054001. 10.1088/1741-2552/ac9643 PMC10353574 36174538

[B36] Verlinden T. J. Rijkers K. Hoogland G. Herrler A. (2016). Morphology of the human cervical vagus nerve: Implications for vagus nerve stimulation treatment. *Acta Neurol. Scand.* 133 173–182. 26190515 10.1111/ane.12462

[B37] Zandstra T. E. Notenboom R. G. E. Wink J. Kies P. Vliegen H. W. Egorova A. D. (2021). Asymmetry and heterogeneity: Part and parcel in cardiac autonomic innervation and function. *Front. Physiol.* 12:665298. 10.3389/fphys.2021.665298 34603069 PMC8481575

